# Conversion of poplar biomass into high-energy density tricyclic sesquiterpene jet fuel blendstocks

**DOI:** 10.1186/s12934-020-01456-4

**Published:** 2020-11-12

**Authors:** Gina M. Geiselman, James Kirby, Alexander Landera, Peter Otoupal, Gabriella Papa, Carolina Barcelos, Eric R. Sundstrom, Lalitendu Das, Harsha D. Magurudeniya, Maren Wehrs, Alberto Rodriguez, Blake A. Simmons, Jon K. Magnuson, Aindrila Mukhopadhyay, Taek Soon Lee, Anthe George, John M. Gladden

**Affiliations:** 1 Joint BioEnergy Institute, Lawrence Berkeley National Laboratory, Emeryville, CA 94608 USA; 2 Biomass Science and Conversion Technology Department, Sandia National Laboratories,, Livermore, CA 94551 USA; 3 Advanced Biofuels and Bioproducts Process Development Unit, Lawrence Berkeley National Laboratory, Emeryville, CA 94608 USA; 4 Biological Systems and Engineering Division, Lawrence Berkeley National Laboratory, Berkeley, CA 94720 USA; 5 Pacific Northwest National Laboratory, Richland, WA 99354 USA; 6 Environmental Genomics and Systems Biology Division, Lawrence Berkeley National Laboratory, Berkeley, CA 94720 USA

**Keywords:** *Rhodotorula toruloides*, Jet fuel, High density, Biofuel, Prespatane, Epi-isozizaene, Pretreatment and saccharification, Poplar

## Abstract

**Background:**

In an effort to ensure future energy security, reduce greenhouse gas emissions and create domestic jobs, the US has invested in technologies to develop sustainable biofuels and bioproducts from renewable carbon sources such as lignocellulosic biomass. Bio-derived jet fuel is of particular interest as aviation is less amenable to electrification compared to other modes of transportation and synthetic biology provides the ability to tailor fuel properties to enhance performance. Specific energy and energy density are important properties in determining the attractiveness of potential bio-derived jet fuels. For example, increased energy content can give the industry options such as longer range, higher load or reduced takeoff weight. Energy-dense sesquiterpenes have been identified as potential next-generation jet fuels that can be renewably produced from lignocellulosic biomass.

**Results:**

We developed a biomass deconstruction and conversion process that enabled the production of two tricyclic sesquiterpenes, epi-isozizaene and prespatane, from the woody biomass poplar using the versatile basidiomycete *Rhodosporidium toruloides*. We demonstrated terpene production at both bench and bioreactor scales, with prespatane titers reaching 1173.6 mg/L when grown in poplar hydrolysate in a 2 L bioreactor. Additionally, we examined the theoretical fuel properties of prespatane and epi-isozizaene in their hydrogenated states as blending options for jet fuel, and compared them to aviation fuel, Jet A.

**Conclusion:**

Our findings indicate that prespatane and epi-isozizaene in their hydrogenated states would be attractive blending options in Jet A or other lower density renewable jet fuels as they would improve viscosity and increase their energy density. Saturated epi-isozizaene and saturated prespatane have energy densities that are 16.6 and 18.8% higher than Jet A, respectively. These results highlight the potential of *R. toruloides* as a production host for the sustainable and scalable production of bio-derived jet fuel blends, and this is the first report of prespatane as an alternative jet fuel.

## Background

New oil discoveries and improved technologies to extract non-traditional oil resources can temporarily increase or maintain the supply of this nonrenewable resource to meet global energy demands [1–3]. However, reliance on fossil fuels is unsustainable in the long term and alternatives must be developed. In an effort to decarbonize transportation, there has been a shift towards electrification, which is already being successfully deployed in road transportation, but is not a viable strategy for other sectors such as aviation. Aviation remains almost completely dependent on non-renewable liquid fuels, yet airline passenger traffic has continued to increase over 5% per year since 2000, accounting for the estimated 31% increase in global petroleum-derived jet fuel consumption that was responsible for almost 1 trillion kg of CO_2_ emissions in 2019 [4–10]. With jet fuel accounting for 9% of the total greenhouse gas emissions from the transportation sector [11, 12], the development of renewable alternatives has become an important priority [5–10].

**Fig. 1:**
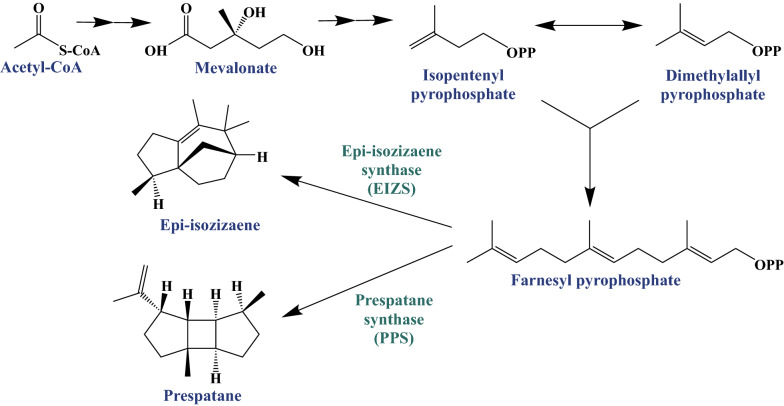
Production of the sesquiterpenes epi-isozizaene and prespatane from acetyl-CoA via the mevalonate pathway. Farnesyl diphosphate cyclization reactions are catalyzed by epi-isozizaene synthase (EIZS) and prespatane synthase (PPS). Both molecules are the major sesquiterpene product of their respective enzymes [18, 21]

Due to their high cetane numbers and energy densities, a number of tricyclic sesquiterpenes have been identified as potential components of next-generation renewable jet fuels [13–15]. A fuel blend based on hydrogenated cedarwood oil (composed primarily of the tricyclic sesquiterpenes thujopsene, ɑ-cedrene, and β-cedrene) was found to have a volumetric net heat of combustion (NHOC) more than 12% higher than conventional jet fuel [13]. In this study, we expand the analysis of this promising category of molecules by investigating two tricyclic sesquiterpenes, epi-isozizaene and prespatane, for their suitability as renewable jet fuel blends. Epi-isozizaene (Fig. 1) is produced naturally by several *Streptomyces* species and initially sparked interest as a candidate jet fuel due to the fact that its specific energy is similar to jet fuel A-1 [16–20]. Prespatane (Fig. 1) is a little-known sesquiterpene produced by the red macroalga, *Laurencia pacifica* [21], that represents a structural class that has not been investigated as a jet fuel. A previous study estimated that saturated terpene jet fuels, such as epi-isozizaane, could be produced from lignocellulose and used as Jet A fuel with a minimum selling price of $0.73–0.91 per liter ($2.75–3.45 per gallon), indicating that there is a real economic potential to use these molecules as an alternative jet fuel when derived from lignocellulose [22].

Lignocellulosic biomass is an excellent carbon source for biorefineries that produce high-volume biofuels and bioproducts because it is the most abundant renewable carbon-source after CO_2_ and is widely available [23]. Generation of jet fuels from lignocellulosic biomass is potentially the best possible mechanism for reducing dependence on petroleum, mitigating greenhouse gas emissions [12] and increasing domestic energy independence [22]. However, in order for this to be accomplished, technologies need to be developed to efficiently and economically deconstruct and convert lignocellulosic biomass [22, 24]. In this study, we demonstrate a process that integrates both deconstruction and conversion of woody lignocellulosic biomass to produce two potential jet fuels.

The oleaginous yeast *Rhodosporidium toruloides* (also known as *Rhodotorula toruloides* [25]) has previously been engineered to produce heterologous isoprenoids from lignocellulosic hydrolysates generated from various plant feedstocks [26–29]. It was used in an integrated separation-free process that combines pretreatment, saccharification, and conversion to produce the D2 diesel replacement bisabolene from sorghum grass [26]. In this study, we explore whether we can expand the use of this process to produce jet fuel from the woody biomass poplar, a promising feedstock for biochemical conversion [30, 31]. Poplar is adaptable to various climates, is easily regenerated by coppicing and has the potential to provide a low-cost feedstock for biorefineries when grown in various scenarios such as short rotation coppice [32].

Finally, in addition to demonstrating the production of epi-isozizaene and prespatane from woody biomass, we sought to determine whether these two sesquiterpenes have properties that are amenable for their use as jet fuels. To do this, we calculated their theoretical properties in their native and fully hydrogenated states and compared them to the known properties of Jet A. That assessment indicated that both sesquiterpenes are indeed good candidates blending into conventional jet fuel. This is the first report of epi-isozizaene and prespatane production in a basidiomycete yeast capable of fully converting lignocellulosic sugars and demonstration of a scalable bioconversion process for converting woody biomass into jet fuel.

## Results and discussion

### Comparison of predicted fuel properties of tricyclic sesquiterpenes with Jet A

The physical properties of aviation fuels and their acceptable ranges are governed by ASTM D1655-19, “Standard Specification for Aviation Turbine Fuels” [33]. These properties include boiling point, viscosity, melting point, energy density, and specific energy. This study modeled these physical properties for the pure molecules and their saturated analogues. The methodologies used are described in the supplementary information and the results are shown in Table 1. Double bonds reduce the stability of jet fuels and are not allowed above ppm levels. Therefore, saturated versions of prespatane and epi-isozizaene were evaluated as aviation fuel components in this study; the unsaturated values are shown for reference.

**Table 1.** Relevant physical properties estimated for epi-isozizaene, prespatane, saturated epi-isozizaene, and saturated prespatane. Methodologies for arriving at these predictions are described in SI

	Epi-isozizaene	Prespatane	Saturated epi-isozizaene	Saturated prespatane	ASTM specification	Median Jet A values*
Liquid density (kg/m^3^)	1000	900	963	966	775–840	810
Boiling point (°C)	273.81	255.58	257.71	261.18	≤300	274.64
Melting point (°C)	− 1.92	1.88	25.79	10	<− 40	− 49.43
Viscosity (mm^2^/s @ − 20 °C)	3.04	4.05	3.17	3.93	<8	4.59
Viscosity (mm^2^/s @ − 40 °C)	5.02	7.09	5.28	6.84	<12	–
Energy density (MJ/L)	39.25	41.42	40.69	41.46	–	34.9
Specific energy (MJ/kg)	42.58	43.06	42.72	43.27	>42.8	43.2

*Volume weighted

In addition to predicting the properties of the neat molecules, a blend model for Jet A was developed. This enabled us to model how the properties of the fuel will vary as the molecules in this study are blended into Jet A. Details of how this blend model was developed can be found in the supplementary information. With this model, estimates of the blending behavior of saturated prespatane and saturated epi-isozizaene were carried out. These calculations focused on viscosity at − 20 and − 40 °C. Additional file 1: Figure S1 shows the results of this work (Additional file 1).

The liquid density in ASTM 1655-19 is measured at 15 °C and has an acceptable range of 775–840 kg/m^3­^ for a drop-in fuel. This liquid density limit is not a safety or operability requirement. It is connected to how fuel level measurements are made in the aircraft, i.e., the incumbent infrastructure. Outside of this range, a fuel would need to be either blended into a base fuel to achieve the required specification or fueling / hardware infrastructure would need to be changed. For epi-isozizaene, saturated epi-isozizaene, prespatane, and saturated prespatane, this value is estimated to be 1000, 963, 900, and 966 kg/m^3^, respectively. For the saturated molecules, these values represent a 7–19% increase over the upper bounds acceptable by ASTM D1655-19. Therefore, for these molecules to be considered “drop-in”, they would have to be considered as blends, with respect to their liquid density. Additional file 1: Figure S2 shows that all molecules can be blended into Jet A at up to ~ 30% (by volume) before reaching the liquid density limit (Additional file 1). Epi-isozizaene can be blended in the largest proportion, reaching the upper limit of 840 kg/m^3^ before the 40% blend level based on the ASTM D1655-19 standard test method.

With regard to viscosity, Jet A at − 20 °C must not exceed 8 mm^2^/s. In addition, there are extended requirements outlined in ASTM D1655-19, which are applicable to fuels containing co-hydroprocessed esters and fatty acids. According to these requirements, at − 40 °C the viscosity cannot exceed 12 mm^2^/s. This metric was included in our study because it is typically included in Jet A requirements [33]. Additional file 1: Figure S3 depicts the temperature dependence of these two blendstocks from − 40 to 40 °C (Additional file 1). Based on these data, both hydrogenated prespatane and epi-isozizaene meet the viscosity requirements as neat fuels. Furthermore, blending these molecules into Jet A would be beneficial by further reducing the viscosity of the final blended fuel.

The maximum acceptable melting point of an aviation fuel is − 40 °C. Table 1 highlights the melting points of epi-isozizaene, prespatane, saturated epi-isozizaene, and saturated prespatane (− 1.92, 1.88, 25.79, and 10 °C, respectively). As neat fuels, they fail to meet the − 40 °C upper limit outlined by ASTM D1655-19. However, the melting point of a blend is highly non-linear and the complex chemical interactions responsible for melting are not easily represented. Further evaluations are required to establish their behavior in blends. However, there is evidence to suggest that by blending into Jet A, the melting point would be reduced.

Table 1 depicts calculations of energy densities and specific energies. For epi-isozizaene, the specific energy was calculated to be 42.58 MJ/kg. This is lower than the Jet A specification minimum of 42.8 MJ/kg by 0.51%. For prespatane, the specific energy was calculated to be 43.06 MJ/kg. This is 0.61% higher than the Jet A specification. By contrast, saturated epi-isozizaene has a specific energy estimate of 42.72 MJ/kg, which is 0.18% lower than the Jet A specification. Saturated prespatane has a specific energy estimate of 43.27 MJ/kg, which is 1.1% higher than the Jet A specification. Jet A does not have a specific energy specification. However, the parameter does impact fuel performance, as higher specific energy fuels allow airplanes to burn less fuel (by weight) to reach their destinations. Jet A has a median energy density of 34.9 MJ/L [34] while saturated epi-isozizaene has a calculated energy density of 40.69 MJ/L, representing a 16.59% increase over the Jet A median value. Saturated prespatane has an energy density estimate of 41.46 MJ/L, representing an 18.79% increase over the Jet A median value. These are substantial increases over the Jet A median energy density value.

### Strain development and optimization for production of epi-isozizaene and prespatane

*R. toruloides* genomic DNA has an overall high GC-content of 62.93% [35], indicating that codon optimization may be particularly important for expression of heterologous genes. Indeed, the majority of studies describing heterologous biofuel production in *R. toruloides* employed codon optimized gene sequences to ensure effective expression [26–29] and comparison to non-optimized genes has not been explored to date. Both codon-optimized and native versions of prespatane synthase from *L. pacifica* (native GC-content: 51%) and epi-isozizaene synthase from *S. coelicolor* A3(2) (native GC-content: 56%) were expressed in *R. toruloides*.

Codon optimization resulted in a 14% increase in GC-content for both genes. Rare codons for *R. toruloides* (those that occur below 20% of frequency expected if there was no codon bias; namely, ATA, CTA, TTA, AGA, and GTA) were present at relatively high frequencies in both native genes. In the native EIZS gene, TTA (13.5%) and GTA (31.8%) were present at a much higher frequency than in *R. toruloides* (0.8 and 4.3%, respectively). In the native PPS gene, ATA (23.5%), CTA (3.3%), TTA (13.3%), AGA (30%), and GTA (10%) were present at a higher frequency than that in *R. toruloides* (3.3, 2.6, 0.8, 3.2, and 4.3%, respectively).

**Fig. 2:**
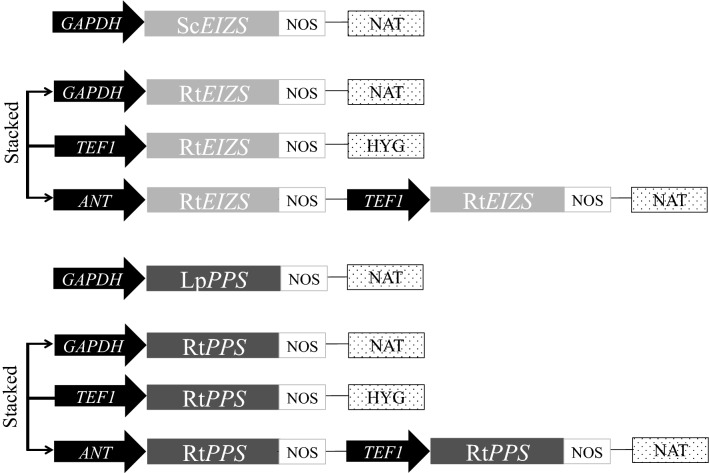
Constructs of relevant plasmids. Arrows labeled “stacked” represent *TEF1*-HYG constructs that were transformed into the highest respective sesquiterpene-producing nourseothricin-resistant (NAT) strains to increase transgene expression. Rt represents *R. toruloides* codon optimized genes while Sc and Lp represent native genes from *S. coelicolor* and *L. pacificia*. NAT and HYG represent expression cassettes conferring resistance to nourseothricin and hygromycin B, respectively, and NOS represents the nopaline synthase terminator

A total of 8 constructs were transformed into *R. toruloides* (Fig. 2 and Table 2) using *Agrobacterium tumefaciens* mediated transformation (ATMT) and 20 individual clones were selected from each transformation for initial screening. Overall, no prespatane or epi-isozizaene was detected in strains harboring native synthases (constructs 1 and 5, respectively). This indicates the importance of codon optimization. In contrast, terpene production in strains harboring the codon optimized genes was appreciable. Interestingly, the highest epi-isozizaene production was observed when a single cassette for EIZS expression was integrated, driven by the *R. toruloides GAPDH* promoter, while prespatane production was highest when two PPS cassettes were integrated, under control of *R. toruloides* promoters *ANT* and *TEF1* (Additional file 1: Figure S4). GC-MS analysis shows that both synthases produced minor terpene products besides the main product (Additional file 1: Table S1), which is typical of terpene synthases.

**Table 2.** Genotypes, features and JBEI registry IDs of plasmid constructs and *R. toruloides *strains investigated in this study

Plasmids	Genotypes/features	Source/references	JBEI registry ID
Construct 1	P_*GAPDH*_-Sc*EIZS*-NAT^R^	This study	JPUB_013517
Construct 2	P_*GAPDH*_-Rt*EIZS*-NAT^R^	This study	JPUB_013519
Construct 3	P_*TEF1*_-Rt*EIZS*-HYG^R^	This study	JPUB_013521
Construct 4	P_*ANT*_-Rt*EIZS*-P_*TEF1*_-Rt*EIZS*-NAT^R^	This study	JPUB_013523
Construct 5	P_*GAPDH*_-Lp*PPS*-NAT^R^	This study	JPUB_013525
Construct 6	P_*GAPDH*_-Rt*PPS*-NAT^R^	This study	JPUB_013527
Construct 7	P_*TEF1*_-Rt*PPS*-HYG^R^	This study	JPUB_013529
Construct 8	P_*ANT*_-Rt*PPS*-P_*TEF1*_-Rt*PPS*-NAT^R^	This study	JPUB_013531
**Strains**			
IFO0880 (WT)	*Rhodosporidium toruloides *strain IFO0880, mating type A2	NBRC culture collection	
EIZS1	IFO0880/P_*TEF1*_-Rt*EIZS*-HYG^R^	This study	JPUB_013534
EIZS2	IFO0880/P_*GAPDH*_-Rt*EIZS*-NAT^R^	This study	JPUB_013532
EIZS3	IFO0880/P_*GAPDH*_-Rt*EIZS*-NAT^R^/P_*TEF1*_-Rt*EIZS*-HYG^R^	This study	JPUB_013533
EIZS4	IFO0880/P_*ANT*_-Rt*EIZS*-P_*TEF1*_-Rt*EIZS*-NAT^R^	This study	JPUB_013535
EIZS5	IFO0880/P_*ANT*_-Rt*EIZS*-P_*TEF1*_-Rt*EIZS*-NAT^R^/P_*TEF1*_-Rt*EIZS*-HYG^R^	This study	JPUB_013536
PPS1	IFO0880/P_*TEF1*_-Rt*PPS*-HYG^R^	This study	JPUB_013539
PPS2	IFO0880/P_*GAPDH*_-Rt*PPS*-NAT^R^	This study	JPUB_013537
PPS3	IFO0880/P_*GAPDH*_-Rt*PPS*-NAT^R^/P_*TEF1*_-Rt*PPS*-HYG^R^	This study	JPUB_013538
PPS4	IFO0880/P_*ANT*_-Rt*PPS*-P_*TEF1*_-Rt*PPS*-NAT^R^	This study	JPUB_013540
PPS5	IFO0880/P_*ANT*_-Rt*PPS*-P_*TEF1*_-Rt*PPS*-NAT^R^/P_*TEF1*_-Rt*PPS*-HYG^R^	This study	JPUB_013541

*GAPDH* glyceraldehyde 3-phosphate dehydrogenase, *TEF1* translational elongation factor, *ANT* adenine nucleotide translocase, Sc*EIZS* epi-isozizaene synthase from *Streptomyces coelicolor* A3(2) (NCBI accession number, WP_011030119.1), Rt*EIZS* epi-isozizaene synthase from *S. coelicolor* A3(2) codon optimized for *R. toruloides*, Lp*PPS* prespatane synthase from *Laurencia pacifica* (ASV63464.1), Rt*PPS* prespatane synthase from *L. pacifica* codon optimized for *R. toruloides*, NAT^R^ nourseothricin resistance; HYG^R^, hygromycin B resistance

**Fig. 3:**
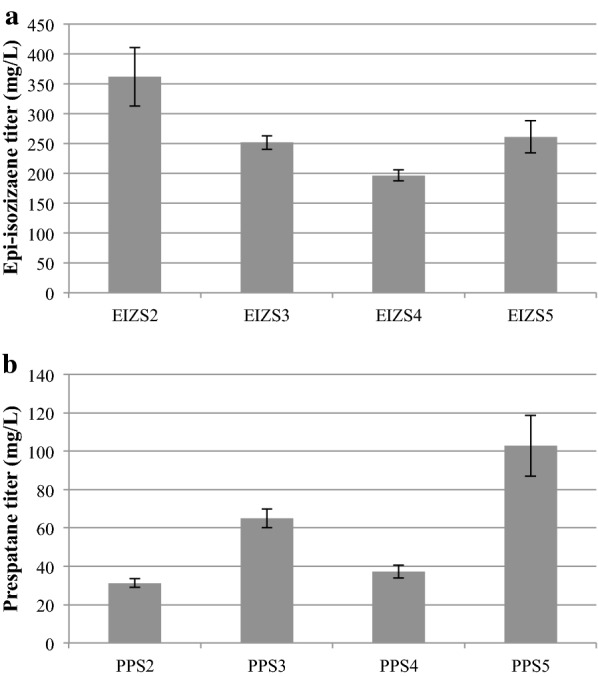
Sesquiterpene titers of highest terpene-producing *R. toruloides* strains from this study. **a** Epi-isozizaene and **b** prespatane titers at day 7 for strains representing genotypes listed in Table 1 that were grown in YPD_10_ with a 20% dodecane overlay. (n = 3, data shown as average ± standard deviation, from a single experiment)

To test whether increasing the copy number of the heterologous gene improves production of the respective sesquiterpene, the highest titer epi-isozizaene (EIZS2 and EIZS4) and prespatane (PPS2 and PPS4) strains were transformed with construct 3 and construct 7, respectively, creating strains EIZS3, EIZS5, PPS3 and PPS5 (Table 2 and Fig. 3). After a second round of transformation, terpene titers increased relative to the parent strain for all but one strain, EIZS2 (362.0 ± 49.4 mg/L), as it remained the highest epi-isozizaene producer. However, prespatane production increased to 102.9 ± 16.0 mg/L with strain PPS5. The best producing strains were selected for further analysis by growth on poplar hydrolysate.

### Optimization of poplar deconstruction

The use of inexpensive renewable carbon sources such as lignocellulose is important for realizing sustainable biofuel production. Previously, Sundstrom et al*.* demonstrated an integrated ionic liquid pretreatment, saccharification and conversion process for the production of the sesquiterpene bisabolene from the grass sorghum using the host *R. toruloides* [26]. In this study, we investigated the suitability of woody biomass as a feedstock in this process for production of the potential jet fuels epi-isozizaene and prespatane using *R. toruloides*. The pretreatment and saccharification of poplar using the ionic liquid (IL) cholinium lysinate was optimized in three consecutive rounds, using the Sundstrom process as a baseline (Table 3). Generally, lowering the biomass loading while increasing the duration of the pretreatment resulted in an increase in saccharification efficiency. Finally, changing the saccharification enzyme cocktail from CTec2/HTec2 to CTec3/HTec3 further improved the process, resulting in yields of 77.0 g/L glucose and 26.6 g/L xylose. In all cases, a dense suspension of fine particles remained in the poplar hydrolysate after saccharification.

**Table 3.** Comparison of pretreatment, enzymatic saccharification, and sugar generation between poplar hydrolysate batches and the sorghum method used previously [26]

	Batch 1	Batch 2	Batch 3	Sundstrom et al. [26]
Pretreatment				
Biomass loading (%)^a^	30	25	25	30
[Ch][Lys]:water	1:09	1:09	1:09	1:09
Temperature (°C)	140	140	140	140
Duration (hour)^a^	1	3	3	1
Enzymatic saccharification				
H_2_SO_4_ pH adjustment to 5 (v/v)	50	50	50	50
Solids loading (w/w)	20	20	20	20
Cellic enzymes^a^	CTec3/HTec3	CTec2/HTec2	CTec3/HTec3	CTec2/HTec2
Enzyme:water	9:01	9:01	9:01	9:01
Temperature (°C)	50	50	50	50
Duration (hour)	72	72	72	72
Sugar				
Glucose (g/L)	46.3	50.1	77	–
Xylose (g/L)	14.2	16.5	26.6	–

^a^Rows indicate variation in biomass loading, pretreatment duration, and enzymes

### Bench-scale optimization of poplar hydrolysates for production of jet fuel blend candidates

Lignocellulosic hydrolysates are deficient in nitrogen, which is an important nutrient for microbial growth and bioconversion. In addition, nitrogen limitation is known to enhance accumulation of storage lipids in oleaginous yeast [36, 37] suggesting that an increase in metabolic flux through acetyl-CoA occurs. This metabolite is also a precursor for isoprenoid biosynthesis and therefore a reduced nitrogen concentration may lead also to increased sesquiterpene production in *R. toruloides* [38]. Therefore, two nitrogen sources were investigated to optimize nitrogen source and concentration for sesquiterpene production in poplar hydrolysate. The strain EIZS2 was used for the optimization. Growth and epi-isozizaene production was quantified for one week in poplar hydrolysate (batch 1: 46.3 ± 2.1 g/L glucose and 14.2 ± 4.7 g/L xylose) supplemented with two different nitrogen sources (Additional file 1: Figure S5). Supplementation with 5 g/L ammonium sulfate led to higher epi-isozizaene production (284.0 ± 28.9 mg/L) compared to 10 g/L yeast extract (138.9 ± 26.4 mg/L). In addition, doubling the concentration of ammonium sulfate provided no additional benefit (266.5 ± 14.5 mg/L). Growth and sugar utilization were similar across all conditions except xylose consumption, which was around 12% lower in the hydrolysate supplemented with yeast extract (77.0 ± 0.8%) and likely contributed to the lower titer. Interestingly, even though the control medium (YPD) had the highest starting glucose concentration (105.8 g/L) and cultivation resulted in 100% glucose utilization, the titer achieved (278.0 ± 20.0 mg/L) was similar to that of the hydrolysate supplemented with ammonium sulfate, suggesting that lignocellulosic hydrolysates can be more efficiently converted into biofuel by *R. toruloides* than rich medium.

**Fig. 4:**
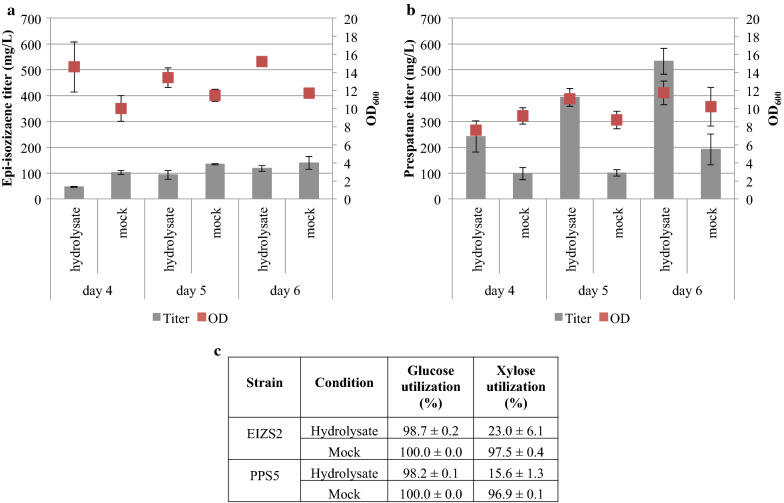
Comparison of terpene production by *R. toruloides* in poplar hydrolysate and mock hydrolysate. Sesquiterpene titers and OD_600_ of strains **a** EIZS2 and **b** PPS5 in filtered batch 2 poplar hydrolysate and a mock hydrolysate with equivalent sugar concentrations, each supplemented with 5 g/L ammonium sulfate. **c** Percent utilization of sugar. (n = 3, data shown as average ± standard deviation, from a single experiment)

The optimized medium composition was used to better characterize the production of the jet fuel candidates epi-isozizaene and prespatane using the high-producing clones EIZS2 and PPS5 in filtered batch 2 poplar hydrolysate (50.1 g/L glucose and 16.5 g/L xylose), which was compared to a mock medium with equivalent concentrations of glucose and xylose. PPS5 produced nearly triple the amount of prespatane in hydrolysate supplemented with 5 g/L ammonium sulfate (532.9 ± 50.3 mg/L) than in mock medium (192.3 ± 59.9 mg/L) (Fig. 4b). While glucose utilization between the two conditions were similar, xylose utilization was significantly lower in hydrolysate (15.9 ± 1.0%) compared to that of mock (96.9 ± 0.1%) (Fig. 4c). These observations are in agreement with previous studies, finding that heterologous isoprenoid production is higher in a lignocellulosic hydrolysate than in an equivalent mock hydrolysate [26]. However, these observations should always be made with the qualifier that it is next to impossible to recreate a mock hydrolysate that perfectly matches the lignocellulosic hydrolysate. Lignocellulosic hydrolysates are compositionally complex and there are likely additional carbon sources, minerals, vitamins, and/or amino acids present in the hydrolysate that could be supplying additional carbon, essential cofactors, and limiting nutrients or may be inducing metabolic shifts that lead to the observed higher productivity (Additional file 1: Table S3). In addition, *R. toruloides* will not grow in a solution of sugars and ammonium sulfate and it requires additional supplementation, further complicating and diverging the comparison. Thus, the comparison between an actual and a mock hydrolysate, while interesting, cannot be considered a direct comparison and a degree of caution should be taken when interpreting these results. Fortunately for *R. toruloides*, it appears to prefer the natural supplements it receives from lignocellulose compared to traditional media supplements, an observation will require extensive future studies to understand.

### Bioreactor scale production of prespatane from poplar using *Rhodosporidium toruloides*

Previous literature reported better performance of *R. toruloides* (engineered to produce α-bisabolene) in corn stover hydrolysate than in defined medium [28]. To investigate the potential of using a woody biomass as a bioconversion feedstock, we attempted to adapt the process developed for grassy biomass to accommodate poplar and identified key parameters that need to be modified to efficiently convert the woody biomass.

An ideal process would utilize hydrolyzed biomass directly in the fermentation process, with no solids separation as this step adds costs and reduces efficiency. This was successfully demonstrated in the aforementioned separation-free process, based on sorghum. However, our initial observations using poplar suggested that the residual particles remaining after saccharification may interfere with fermentation. To better understand this observation, we chose to investigate three different configurations to determine the impact of the biomass residue on growth and production. Filtered, unfiltered and mock poplar hydrolysates from batch 3 (77.0 g/L glucose and 26.6 g/L xylose) were tested in separate fermenters. Since it produced the highest sesquiterpene titer in the bench scale studies, the prespatane strain PPS5 was chosen to investigate production at a larger 2 L scale. An initial test fermentation with hydrolysate suggested that magnesium and phosphate were rapidly depleted, so these three test runs were supplemented with 0.5 g/L magnesium sulfate and 1 g/L potassium phosphate to avoid a possible nutrient deficiency (Additional file 1: Figure S6).

**Fig. 5:**
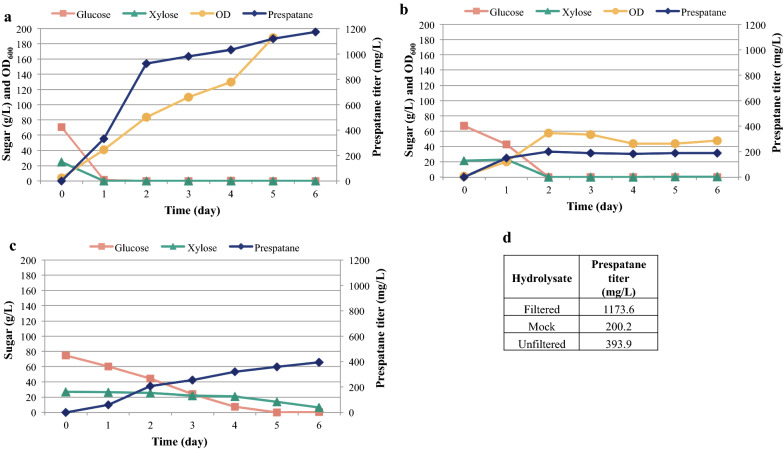
Production of prespatane from poplar hydrolysate by *R. toruloides* at 2 L scale. Sugar concentrations, OD_600_, and prespatane titers for 2 L bioreactor runs with PPS5 in **a** filtered **b** mock and **c** unfiltered poplar hydrolysate from batch 3. **d** Highest prespatane titer achieved in each case

Of the three test cultivations, the highest prespatane titer was achieved when strain PPS5 was grown in filtered hydrolysate (Fig. 5a), reaching prespatane titers of 1173.6 mg/L. While both glucose and xylose had been almost completely utilized by 24 h, the prespatane titer continued to increase over the course of six days. The cell density (OD_600_) also consistently increased, reaching 188 on day five. Similar to the previous experiment, the titer achieved in the mock hydrolysate remained relatively low at 200.2 mg/L, sugar utilization was twice as slow as the filtered hydrolysate and the cell density was 3–fourfold lower (Fig. 5b). Finally, growth in the unfiltered hydrolysate yielded a titer of 358.2 mg/L, which is significantly lower than the filtered but higher than the mock hydrolysate (Fig. 5c). However, the sugar utilization rate was significantly lower than the other two cultivations, indicating the particles are interfering with either sugar uptake or cell growth. Interestingly, a spike of lactic acid appeared between day 3 and 4, indicating possible contamination by a lactic acid bacterium. The lactic acid was consumed by *R. toruloides* in the subsequent days so no carbon was lost from the portion of sugar converted to lactic acid (Additional file 1: Figure S7). While it is possible that lactic acid could have affected prespatane titers, this acid was also observed in the previous sorghum study where the lactic acid appeared to have no impact on the overall titer of bisabolene. Therefore, the lower prespatane titers observed in unfiltered hydrolysate is likely due to the residual poplar particles present in the hydrolysate.

### Yeast viability in presence of poplar solids

Visual inspection of the poplar particles remaining after saccharification indicated that they were smaller and more dense than the equivalent residual sorghum particles. One possibility is that the smaller particles were having a negative impact on cell growth in a bioreactor with high agitation where the particles will be continually colliding with the cells. To test this, cell density in filtered and unfiltered poplar hydrolysate was counted using a hemocytometer. There was a significant difference in titer between filtered and unfiltered poplar hydrolysate (*P* = 0.0003). After six days of growth, the prespatane titer in filtered hydrolysate reached 292.8 ± 12.8 mg/L with all sugar consumed, while production in the unfiltered hydrolysate reached 63.9 ± 11.8 mg/L with minimal sugar utilization (Additional file 1: Figure S8). Cell counts in unfiltered hydrolysate (4.5 × 10^8^ ± 5.7 × 10^7^) were significantly lower (P = 0.04) than in the filtered hydrolysate (3.0 × 10^9^ ± 9.3 × 10^8^). This suggests that the poplar particles produced after pretreatment and saccharification affect the growth and/or viability of *R. toruloides*. In contrast, *R. toruloides* grown in unfiltered hydrolysate derived from sorghum was able to produce high titers of bisabolene [26]. These results suggest that further process optimization will be required to use unfiltered poplar hydrolysates, such as reduced agitation or a higher inoculation of *R. toruloides* cells.

## Conclusion

The integration of *R. toruloides* strains engineered to produce the tricyclic sesquiterpenes epi-isozizaene and prespatane into a woody biomass lignocellulosic conversion process holds great promise for the production of renewable jet fuel. This is the first study to investigate prespatane as a jet fuel and outlines the first step on a roadmap toward establishing a sustainable jet fuel blend generated by bioconversion of lignocellulosic feedstocks. Various theoretical fuel properties of epi-isozizaene and prespatane and their hydrogenated states were predicted in this study and compared with those of Jet A. For example, specific energy and energy density are important properties in determining the attractiveness of potential bio-derived jet fuels; increased energy content can give the industry options such as longer range, higher load or reduced takeoff weight. These and other sesquiterpenes were shown to have high cetane numbers and energy densities, making them good candidates for the next-generation of jet fuel. Although the hydrogenated sesquiterpenes do not meet all of the ASTM standards for jet fuel, such as melting point, their properties suggest suitability for blending and their high energy densities will likely confer performance advantages.

To improve the sustainability of biofuel production, we demonstrated that lignocellulosic biomass can be used as an inexpensive carbon source for bioconversion using *R. toruloides* and actually outperforms pure sugars in the context of terpene production. Specifically, we demonstrate that *R. toruloides* can convert poplar lignocellulosic hydrolysates into epi-isozizaene and prespatane. Furthermore, scalability was demonstrated when improved performance was observed at a 2 L scale, reaching 1173.6 mg/L. Prespatane titers improved when transitioning from mock and unfiltered hydrolysate to filtered IL hydrolysate and full consumption of the major carbon sources was observed within 24 h, demonstrating the promise of *R. toruloides* as production host for efficient conversion of IL pretreated biomass. With further process intensification and optimization, our process presents a promising new approach towards commercial production of lignocellulosic jet fuel blends.

## Materials and methods

### Theory prediction methodology

For Table 1, boiling point predictions were made using the Stein and Brown method [39], which is a variant of the more widely known Joback and Reid method [40]. In the Stein and Brown method, the Joback and Reid result is augmented using the following equation:

$${T}_{b}\left(corr\right)= {T}_{b}-94.84+0.5577{T}_{b}-0.0007705{T}_{b}^{2}, $$

where ${T}_{b}$ is the Joback Reid result and ${T}_{b}(corr)$ is the Stein and Brown result. The method, using a dataset of 6584 compounds, has an average absolute error of 20.4 K and an average percent error of 4.3%. Equation of State (EoS) modeling requires several basic inputs, one of which is the ideal heat capacity (Cp,_ideal_). The DIPPR (Design Institute for Physical Properties) database is a repository that contains these inputs for a large array of molecules. However, when these inputs are not available, their estimates can be obtained. Cp,_ideal_ was obtained by calculating the optimal geometry of the target molecule and performing a subsequent frequency analysis. The frequency analysis acts as inputs to established statistical thermodynamics equations, which relate frequencies to Cp,_ideal_. The program thermo.pl was used to convert frequencies to Cp,_ideal_ [41]. The geometry optimization/frequency calculation was performed using the B3LYP/6-311G** method [42, 43] and Cp,_ideal_ was calculated from 0 to 1000 K. The resulting Cp,_ideal_ values were then fitted to a 4^th^-order polynomial and used as an input to the Multiflash thermodynamic modeling program [44]. Also, the critical thermodynamic values (T_c_, P_c_) are required as input. They were obtained using the group contribution method as outlined by Marrero et al. [45]. The standard deviations are 6.99 K and 1.39 bar, respectively, and therefore yield accurate estimates of T_c_ and P_c_. These parameters were used to estimate liquid densities. Liquid viscosity was calculated using both the SuperTRAPP and the Pedersen equation. These are two corresponding states methods, which yield accurate viscosities for both pure and complex mixtures, applicable to the oil and gas industry [46]. A description of the SUPERTRAPP method and the full Pedersen equation is listed below. The SUPERTRAPP viscosity model is an extended corresponding states method and only works for liquid mixtures. It uses a combination of the viscosity of a reference fluid (in this case propane), its critical thermodynamics properties, and those of the pure/mixture fluid in order to obtain accurate viscosity estimates. The SUPERTRAPP method can be described by the following equation:

$$\eta \left(T, \rho \right)= {\eta }_{ref}\left(\frac{T}{g}, \rho h\right)\left[\frac{{M}^\frac{1}{2}}{{M}_{ref}^\frac{1}{2}}\right]{g}^\frac{1}{2}{h}^{\frac{-2}{3}}{X}_{\eta },$$

where Xη is a correction factor for noncorrespondence, M is the molar mass, η is the viscosity, and the subscript, ref, refers to the reference fluid. g and h are variables dependent on the critical thermodynamic properties and can be calculated using the equation from Ely and Hanley [47]. The Pedersen equation is listed below:

$${\eta }_{L}\left(P, T\right)=\left(\frac{{T}_{C}^{-\frac{1}{6}}}{{T}_{cr}^{-\frac{1}{6}}}\right)\left(\frac{{P}_{c}^\frac{2}{3}}{{P}_{cr}^\frac{2}{3}}\right)\left(\frac{{MW}^\frac{1}{2}}{{MW}_{r}^\frac{1}{2}}\right)\left(\frac{\alpha }{{\alpha }_{r}}\right){\eta }_{r}({P}_{r}, {T}_{r}),$$

where ${T}_{c}$ is the critical temperature in Kelvin, ${P}_{c}$ is the critical pressure in bar, $MW$ is the molecular weight, η is the viscosity, and the subscript ‘r’ refers to the reference fluid.

$$ a = 1.000 + 7.387x^{-3} \rho _{r}^{{1.847}} MW^{{0.5173}}  $$

$${a}_{r}=1.000+0.031{\rho }_{r}^{1.847},$$

where *ρ*_*r*_ is the reduced density of the reference fluid, in this case methane. These models are not dependent on an EoS but rather the critical thermodynamic properties of the target molecule/mixture as well as the reference fluid.

Additionally, a blend model for a conventional jet fuel was developed. The jet fuel modeled is an A-2 POSF 10325 Jet A fuel. The blend model was developed by utilizing a published GCxGC analysis and incorporating those chemical species which make more than 1 vol% contribution to the fuel [48]. Due to limitations in the GCxGC analysis, only the general type of molecule and the extent to which it is present are known. Therefore, a reasonable assumption as to the type of molecules present needs to be made. For example, all alkylbenzenes were assumed to be n-alkylbenzenes. Di-aromatics were fully represented by naphthalene. Cycloaromatics were modeled as alkyltetralins. Isoparaffins were modeled as 2-methylalkanes. Monocycloparaffins were modeled as n-alkylcyclohexanes, and dicycloparaffins were represented by cis-decalin. Tricycloparaffins, making only trace contributions, were excluded. Viscosity curves of the Jet A blend model, as a function of temperature, were calculated using both the SUPERTRAPP and Pedersen method, and compared to experimental data (Additional file 1: Figure S9). The components of the blend model were also used to access the performance of the SUPERTRAPP and Pedersen method in modeling viscosity. Details of these calculations can be found in Table S2 (Additional file 1). The SUPERTRAPP method is statistically superior to the Pedersen method. Therefore, the viscosity curves of saturated prespatane and saturated epi-isozizaene, blended into our blend model were calculated using the SUPERTRAPP method.

Energy density and Specific energy calculations were performed using ab-initio calculations. Initially, a geometry optimization of the target molecule is performed using the B3LYP/6-311G** method. Frequencies were calculated at the same level of theory. All real and positive valued frequencies are evidence that the optimized geometry is a minima on the Potential Energy Surface (PES). Once an optimized geometry is obtained, a single point ab-initio calculation is performed using the CBS-QB3 (Complete Basis Set) method. In a CBS method, more accurate energetics are obtained by performing a series of single point energy calculations. The resulting energies of these calculations are used as inputs in extrapolative equations. In a test of several hundred molecules in the G2/97 test set, the maximum average deviation in energy of the CBS-QB3 method is 3.63 kJ/mole [49]. In order to calculate the Heat of Combustion (HOC) of a target molecule, the CBS-QB3 method was run for O_2_, CO_2_, H_2_O, and the target molecule. From the CBS-QB3 method, the Heat of Combustion (HOC) is obtained by noting the balanced chemical equation for combustion, and utilizing the following equation:

$$HOC=\left(\sum_{prod}{H}^{o}-\sum_{react}{H}^{o}\right)+HOV,$$

where HOV is the enthalpy of vaporization. Once the HOC is known, the Specific energy and the Energy density can be obtained by using the molecular weight and the liquid density, respectively. All ab-initio calculations were run using the Gaussian 09 computational suite [50]. EoS calculations were carried out using the Multiflash program [44].

### Plasmid design and construction

Plasmids and strains used in this study can be found in Table 2, and are also available through Joint BioEnergy Institute Strain Registry (https://public-registry.jbei.org/ [51]) and are available upon request. Codon optimization, gene synthesis, and plasmid construction was performed by Genscript (Piscataway, NJ). Overall, two sets of four plasmids were designed and tested for the expression of two heterologous enzymes to produce jet fuel: the epi-isozizaene synthase (EIZS) from *Streptomyces coelicolor* A3(2) (NCBI accession number, WP_011030119.1) and the prespatane synthase (PPS) from *Laurencia pacifica* (ASV63464.1). An overview of the plasmids used in this study is shown in Fig. 2. Of those constructs, one contained the native enzyme sequence (Sc*EIZS* and Lp*PPS,* respectively) while the other three contained sequences codon optimized for expression in *R. toruloides* (Rt*EIZS* and Rt*PPS*). Codon optimization was based on a custom IFO0880 codon usage table (https://genome.jgi.doe.gov/Rhoto_IFO0880_3/Rhoto_IFO0880_3.home.html). The promoters *GAPDH*, *TEF1* and *ANT* were used to drive the expression of the heterologous gene. One design had two gene copies, driven by both *ANT* and *TEF1*, respectively. Constructs were synthesized and inserted into the ATMT plasmid pGI2 [52] using the EcoRV restriction sites. The pGI2-derived plasmids were introduced into *R. toruloides* recipient strains by ATMT as previously described [29, 53].

### Transformation and screening of *R. toruloides*

ATMT with strain EHA 105 was performed using *R. toruloides* IFO0880, previously described [53]. Twenty random transformants of each construct were randomly selected and cultivated in 0.5 mL Difco YPD (yeast extract 10 g/L, peptone 20 g/L, and glucose 20 g/L) (VWR, 90,003–284, Radnor, PA) in a 96-well plate (Corning, 3960, Corning, NY) with gas-permeable sealing film (m2p-labs, F-GP, Baesweiler, Germany) for 24 h, shaking on a Multitron (INFORS HT, I10003, Bottmingen, Switzerland) at 31 °C, 1,000 rpm and 70% humidity. The following day, 50 μl of the saturated culture was transferred into 950 μl YPD_10_ (YPD containing 100 g/L of glucose) in a 48-well flower plate (m2p-labs, M2P-48-B). 20% dodecane overlay with an internal standard (200 mg/L pentadecane) was added to the production cultures (Sigma-Aldrich, 76,510, St. Louis, MO). After 7 days of cultivation, at 30 °C, 1,000 rpm, with 70% humidity in the Multitron shaker, the production cultures were centrifuged (21,130 × *g*, 5 min) to separate the overlay from the cultivation media. Centrifugation was in an Eppendorf 5424 Microcentrifuge (Eppendorf AG, 022620428, Hamburg, Germany).

### Quantification of sesquiterpenes

The highest producing clone of each construct was identified (Additional file 1: Figure S4). The plasmid containing the HYG selection marker was transformed into the respective highest producing clone with NAT resistance (i.e., stacking). The highest producing stacked strains were identified and grown in parallel with the original parent strain to confirm titer differences (Fig. 3). Ethyl acetate or dodecane were used to make dilutions (1:10, 1:50, 1:100, 1:125, 1:200, and 1:250) of the overlay with a total volume of 200 μL or 600 μL. The internal standard pentadecane (250 mg/L) was used in the overlay at the beginning of the experiment. Caryophyllene (40 mg/L) and *ent*-kaurene (40 mg/L) were used as internal standards during dilution. Titer was determined using a conversion factor with pure bisabolene standards. The conversion factors were calculated by comparing SIM and SCAN corrected peak area of bisabolene to that of epi-isozizaene and prespatane. The diluted overlay samples were analyzed by gas chromatography—mass spectrometry (GC-MS) using an Agilent 6890 Plus gas chromatograph (Agilent Technologies, G1530A, Santa Clara, CA) operating with an Agilent 5973 Network mass spectrometer (Agilent Technologies, G1099A). 1 μL of each sample was injected by a CombiPal autosampler (CTC Analytics, MXY 02-00B, Zwingen, Switzerland). Analytes were separated on a DB-5MS column (30 m long, 0.25 mm internal diameter, 0.25 μm film thickness, Agilent Technologies, 122–5532) using the following oven parameters: hold for 0.75 min at an initial temperature 100 °C, followed by a temperature ramp of 40 °C /min to 300 °C. The mass spectrometer was operated in selected ion mode, with target ions (m/z) of 71, 85, 119, 161, 189 and 204. Analysis was performed on Enhanced ChemStation (Agilent Technologies, MSD Chemstation E.02.00.493).

### Poplar pretreatment and saccharification

**Fig. 6:**
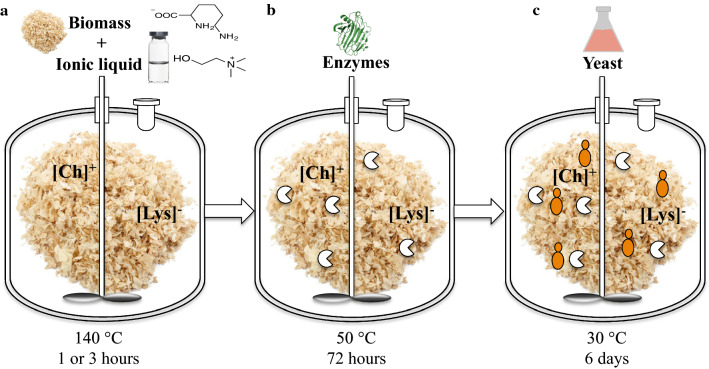
The pretreatment process for conversion of milled poplar wood into terpenes. **a** For pretreatment, the reactor contained 25 or 30% biomass and 10% cholinium lysinate ([Ch]^+^ [Lys]^−^), heated to 140 °C and stirred for either one or three hours. **b** Sulfuric acid 50% (v/v) was used to adjust the pH to between 4.8 and 5. The enzymes CTec3:HTec3 (or CTec2:HTec2) in a ratio of 9:1 (v/v) were added at 30 mg per gram of untreated biomass. The reactor was heated to 50 °C and stirred for 72 h. **c** *R. toruloides* was then added and grown at 30 °C for six days. In some cases, the hydrolysate was filtered beforehand

The process was performed as described by Sundstrom et al. with minor modifications (Fig. 6 and Table 3) [26]. Poplar, with the biomass composition of 42.6% (w/w) glucan and 15.6% (w/w) xylan was used as feedstock. The ionic liquid pretreatment and enzymatic hydrolysis were conducted in an automated 1 L Parr reactor system (Parr Instrument Company, Moline, IL, USA). 30% biomass loading was achieved by using 30 g of biomass in 10:90 [Ch][Lys]: water. The pretreatment was carried out at 140 °C for 1 h with stirring at 90 rpm powered by a 4875 process controller using three-arm, self centering anchor with PTFE wiper blades. After pretreatment, the pH was adjusted to 5 by adding 1.3 mL H_2_SO_4_ at 50% v/v (i.e., 0.3 mol) and the IL–treated biomass was diluted to achieve a solids loading of 20% w/w. The accessibility of enzymes to cellulose and hemicellulose in the poplar was quantified by the yield of sugars (glucose and xylose) released during enzymatic hydrolysis. The cellulase and hemicellulase Cellic® complex CTec3/HTec3 (9:1) were used at loading of 30 mg protein/ g of biomass. The reaction was carried out for 72 h at 50 °C and 90 rpm agitation. The supernatant was analyzed by HPLC for monosaccharide detection [26]. Enzymatic digestibility was defined as the glucose yield based on the maximum potential glucose from glucan in biomass. In the calculation of cellulose conversion to glucose, it was considered cellulose: glucose ratio of 1:1.11 [54]. Overall, three individual batches of poplar hydrolysate were prepared with minor modifications (Table 3).

### Bench-scale nitrogen source optimization: conversion of poplar hydrolysate into jet fuel candidates

Batch 1 poplar hydrolysate was filtered (0.2 μm, VWR, 97,066–212) and supplemented with two nitrogen sources, summarized in Figure S5 (Additional file 1). Those include ammonium sulfate 5 g/L and 10 g/L, and yeast extract 10 g/L. pH was adjusted to 7.5. The control medium was yeast extract 10 g/L, peptone 20 g/L, and glucose 105.8 g/L.

Using triplicate test tubes, the highest epi-isozizaene strain, EIZS2 (JPUB_013532), was grown in 5 mL of supplemented hydrolysate. To collect the sesquiterpene throughout the growth experiment, a 20% dodecane overlay was added. Optical density at 600 nm (OD_600_), sugar and sesquiterpene titer were measured after seven days growth in a 48-well flower plate (Additional file 1: Figure S5). Samples were diluted 1:10 in HPLC grade water (Honeywell, AH365-4, Charlotte, NC) and starting sugars 1:50. Sugars, organic acids and furfurals were quantified by HPLC using an Agilent Technologies 1200 series instrument equipped with an Aminex HPX-87H column (BioRad Laboratories, USA) and a refractive index detector, kept at 60 ºC and 35 ºC, respectively, during analysis (Additional file 1: Figure S5B). The mobile phase was 4 mM sulfuric acid with a flow rate of 0.6 mL/min. 5 μL sample injection volumes were used. Aromatic compounds were tracked by HPLC using an Agilent Technologies 1260 series instrument equipped with an Eclipse Plus Phenyl-hexyl column (250 mm length, 4.6 mm diameter, 5 µm particle size; Agilent Technologies) that was kept at 50 ºC, and using an injection volume of 5 µL. The mobile phase was composed of 10 mM ammonium acetate in water (solvent A) and 10 mM ammonium acetate in acetonitrile 90% (solvent B), prepared from a stock solution of 100 mM ammonium acetate and 0.7% formic acid in water. The following mobile phase gradient profile was used: 30% B (0 min; 0.5 mL/min), 80% B (12 min; 0.5 mL/min), 100% B (12.1 min; 0.5 mL/min), 100% B (12.6 min; 1 mL/min), 30% B (12.8 min; 1 mL/min), 30% B (15.6 min; 1 mL/min). Prior to analysis, samples were filtered using 0.45 µm nylon centrifuge filters. Concentrations were calculated by comparison of the resulting peak areas to calibration curves made with pure standards.

### Determining fermentation strain

After 0.2 μm filtration, the hydrolysate from batch 2 was supplemented with the optimal nitrogen source, ammonium sulfate 5 g/L. Using triplicate test tubes, the top strains PPS5 (JPUB_013541) and EIZS2 were grown in 5 mL of the supplemented batch 2 hydrolysate. A mock medium (yeast extract 10 g/L, peptone 20 g/L, glucose 50.1 g/L, and xylose 16.5 g/L) was used as a control with the same starting concentration of glucose and xylose as the batch 2 hydrolysate. A twenty-percent dodecane overlay was used. OD_600_, sugar and sesquiterpene titer were collected daily between day 4 and 6 (Fig. 4).

### Hydrolysate preparation for bioreactor run

Batch 3 poplar hydrolysate was prepared with CTec3 (protein concentration: 107.7 ± 2.1 mg/mL) and hemicellulase HTec3 (protein concentration: 80.4 ± 5.4 mg/mL) complex for enzymatic hydrolysis (Table 3). They were loaded at a fixed ratio based on the initial biomass content (27 mg CTec3/g biomass, 3 mg HTec3/g biomass). After 72 h of saccharification the poplar hydrolysate was collected for fermentations experiments carried out using unfiltered hydrolysate and filtered hydrolysate (without solids). The filtered batch 3 hydrolysate was prepared by filtering through a 0.7 μm glass fiber filter (Whatman, Maidstone, UK) and sterilized via 0.2 μm filtration. The hydrolysate contained concentrations of 77 g/L glucose (90.3% yield), 26.6 g/L xylose (85.5% yield), and 11.9 g/L acetic acid. The composition of the mock hydrolysate was designed to closely match the sugar concentration of poplar hydrolysate. It consisted of yeast extract (10 g/L), peptone (20 g/L), glucose (77 g/L) and xylose (26.6 g/L).

### Seed cultures

The highest producing prespatane strain, PPS5, was selected for fermentation (Fig. 5). Cell growth during seed culture was performed in three steps. First, cells were cultured in 5 mL YPD media. Then, cells were transferred to 50 mL YPD media and 50 mL of liquid media containing 25% (v/v) poplar hydrolysate and 75% (v/v) YPD. After this step, cells grown in 25% hydrolysate were used to inoculate a 50:50 mixture of YPD with hydrolysate and subsequently used to inoculate the fermenters with hydrolysate. Furthermore, cells grown in 50 mL YPD were used to inoculate the fermenter with mock hydrolysate. In all steps, inoculum was 10% (v/v) and the cells were incubated at 30 °C, 200 rpm for 24 h.

### 2 L bioreactor fermentations

Batch fermentations were performed in 2 L Sartorius fermenters (Sartorius Stedim, Göttingen, Germany) using batch 3 filtered and unfiltered poplar hydrolysate and a mock hydrolysate. One tank was batched with 900 mL unfiltered hydrolysate, one tank contained 787 mL of filtered hydrolysate (considering 12.6% the solid content of the hydrolysate) and the third tank contained 787 mL of mock hydrolysate. For all experiments, 10% (v/v) inoculum and 20% (v/v) dodecane, containing 1 g/L pentadecane as internal standard, were added aseptically into the fermenters in the beginning of the process. Additionally, all reactors were supplemented with ammonium sulfate, magnesium sulfate and potassium dihydrogen phosphate to a final concentration of 5, 0.5 and 1 g/L, respectively.

Unfiltered hydrolysate was pasteurized at 80 °C for 1 h and all the other components were filtered sterilized (0.2 μm pore size filters). To prevent bacterial contamination, 1 mL of 30% (w/v) cefotaxime was added to the batch medium. Fermenters were controlled at 30 °C and initial pH was adjusted to pH 7 and controlled at pH 5 with 2 N NaOH. Dissolved oxygen was cascade-controlled at 20% via agitation (500–1200 rpm) and air flow (0.5–1.5 LPM). Samples were taken in regular intervals and centrifuged to separate aqueous and solvent fraction.

### Filtered vs unfiltered poplar hydrolysate

Small scale comparison of filtered and unfiltered poplar hydrolysate was performed with two to three replicates. Prespatane titer, cell count, cellular morphology, and sugars were measured. Seed cultures, antibiotics, overlay, nitrogen sources, and inoculum (v/v) were the same as the bioreactor fermentation. 125 mL baffled flasks were used with 17 mL hydrolysate, 1 mL 20X nitrogen sources, and 2 mL seed culture. Cells were incubated at 30 °C, 200 rpm for six days. Samples were taken daily.

To fix cells for microscopy, 90 μL were combined with 10 μL of 37% formaldehyde solution (Sigma-Aldrich, 252549) and incubated for 15 min at room temperature. 1 min of 8000 rpm centrifugation occurred, supernatant was removed, and cells were resuspended with 100 μL of 0.1 M potassium phosphate (Sigma-Aldrich, P3786). Samples were stored at 4 °C. 20 μL of cells were stained with 1 μL 10% potassium hydroxide (Sigma-Aldrich, 306568) and 1 μL of calcofluor white (Sigma-Aldrich, 18909) in 78 μL phosphate buffered saline (PBS). Cell count was determined by a hemocytometer.

## Supplementary information

**Additional file 1**: **Table S1**. All metabolites detected by GC-MS from (A) prespatane synthase and (B) epi-isozizaene synthase expressed in *R. toruloides.* Relative abundances were approximated by corrected peak area. Molecules have not been confirmed with standards and tentative molecule assignments are based on mass spectrum matches. **Table S2.** Relative reliability of the viscosity methods SUPERTRAPP and Pedersen (%AAD, Absolute Average Deviation) for molecules included in the blend model. **Table S3.** Concentration of compounds tracked by HPLC in batch 2 poplar hydrolysate supplemented with 5 g/L ammonium sulfate. **Figure S1.** Viscosity blending behavior of saturated prespatane and saturated epi-isozizaene at **a** − 20 °C and **b** − 40 °C. **Figure S2.** Liquid density blending behavior of saturated prespatane and saturated epi-isozizaene at 15 °C. **Figure S3.** Viscosities of saturated prespatane and saturated epi-isozizaene, in the temperature range of − 40–40 °C. **Figure S4.** Sesquiterpene titers of the highest terpene producing strain for each construct shown in Fig. 2 before stacking of HYG and NAT constructs. **Figure S5.** Nitrogen source supplementation comparisons in poplar hydrolysate. **Figure S6.** The initial 2 L fermentation run resulted in a low prespatane titer, which was attributed to a possible magnesium and phosphate deficiency. **Figure S7.** Organic acids from PPS5 fermentation with unfiltered hydrolysate batch 3. **Figure S8.** Prespatane production by PPS5 grown in filtered and unfiltered poplar hydrolysate. **Figure S9.** Validation of a viscosity model for Jet A.

## Data Availability

All data generated or analyzed during this study are included in this published article.
