# Supplementary material for: Conversion of poplar biomass into high-energy density tricyclic sesquiterpene jet fuel blendstocks
Source: Microb Cell Fact. 2020 Nov 12;19:208. doi: 10.1186/s12934-020-01456-4 (PMC7659065; doi:10.1186/s12934-020-01456-4)
Supplement: Supplementary file 1 — Additional file 1: Table S1. All metabolites detected by GC-MS from (A) prespatane synthase and (B) epi-isozizaene synthase expressed in R. toruloides. Relative abundances were approximated by corrected peak area. Molecules have not been confirmed with standards and tentative molecule assignments are based on mass spectrum matches. Table S2. Relative reliability of the viscosity methods SUPERTRAPP and Pedersen (%AAD, Absolute Average Deviation) for molecules included in the blend model. Table S3. Concentration of compounds tracked by HPLC in batch 2 poplar hydrolysate supplemented with 5 g/L ammonium sulfate. Figure S1. Viscosity blending behavior of saturated prespatane and saturated epi-isozizaene at a − 20 °C and b − 40 °C. Figure S2. Liquid density blending behavior of saturated prespatane and saturated epi-isozizaene at 15 °C. Figure S3. Viscosities of saturated prespatane and saturated epi-isozizaene, in the temperature range of − 40–40 °C. Figure S4. Sesquiterpene titers of the highest terpene producing strain for each construct shown in Fig. 2 before stacking of HYG and NAT constructs. Figure S5. Nitrogen source supplementation comparisons in poplar hydrolysate. Figure S6. The initial 2 L fermentation run resulted in a low prespatane titer, which was attributed to a possible magnesium and phosphate deficiency. Figure S7. Organic acids from PPS5 fermentation with unfiltered hydrolysate batch 3. Figure S8. Prespatane production by PPS5 grown in filtered and unfiltered poplar hydrolysate. Figure S9. Validation of a viscosity model for Jet A. [file 12934_2020_1456_MOESM1_ESM.docx]

**Additional file 1**

Conversion of Poplar Biomass into High-Energy Density Tricyclic Sesquiterpene Jet Fuel Blendstocks

Gina M. Geiselman^a,b^, James Kirby^a,b^, Alexander Landera^b^, Peter Otoupal^a,b^, Gabriella Papa^c,d^, Carolina Barcelos^c,d^, Eric R. Sundstrom^c,d^, Lalitendu Das^a,b^, Harsha D. Magurudeniya^a,b^, Maren Wehrs^a,d^, Alberto Rodriguez^a,b^, Blake A. Simmons^a,d^, Jon K. Magnuson^e^, Aindrila Mukhopadhyay^a,d,f^, Taek Soon Lee^a,d^, Anthe George^a,b^, and John M. Gladden^a,b *^

^a^Joint BioEnergy Institute, Lawrence Berkeley National Laboratory, Emeryville, CA 94608, USA

^b^Biomass Science and Conversion Technology Department, Sandia National Laboratories, Livermore, CA 94551, USA

^c^Advanced Biofuels and Bioproducts Process Development Unit, Lawrence Berkeley National Laboratory, Emeryville, CA 94608, USA

^d^Biological Systems and Engineering Division, Lawrence Berkeley National Laboratory, Berkeley, CA 94720, USA

^e^Pacific Northwest National Laboratory, Richland, WA 99354, USA

^f^Environmental Genomics and Systems Biology Division, Lawrence Berkeley National Laboratory, Berkeley, CA 94720, USA

^*^Corresponding author. Tel. +1 (510) 495-2490

Email address: (John M. Gladden) jmgladden@lbl.gov

**A B**

| **Molecule** | **Percentage (%)** |
| --- | --- |
| prespatane | 85.6 |
| bicyclo[4.3.0]nonane, 7-methylene-2,4,4-trimethyl-2-vinyl or (+)-valencene | 8.1 |
| γ-gurjunene | 3.7 |
| ɑ-guaiene or ɑ-selinene | 0.8 |
| γ-elemene | 0.7 |
| (+)-ledene | 0.6 |
| β-guaiene | 0.4 |
| viridiflorene | 0.1 |

| **Molecule** | **Percentage (%)** |
| --- | --- |
| epi-isozizaene | 92.4 |
| 26.85 Khusimene (=zizaene) | 3.7 |
| cedrene or (Z)-β-farnesene | 2.4 |
| ɑ-cedrene | 0.5 |
| ɑ-longipinene or 30.03 bisaboline ((E)-γ)) | 0.5 |
| farnesyl acetone | 0.5 |

**Table S1**. All metabolites detected by GC-MS from (**A**) prespatane synthase and (**B**) epi-isozizaene synthase expressed in *R. toruloides.* Relative abundances were approximated by corrected peak area. Molecules have not been confirmed with standards and tentative molecule assignments are based on mass spectrum matches.

| **Alkylbenzenes** | | | | |
| --- | --- | --- | --- | --- |
| Chemical name | Temperature range (^o^C) | Number of points | SUPERTRAPP (%AAD) | Pedersen (%AAD) |
| ethylbenzene | -40 to 40 | 18 | 3.8 | 36.2 |
| propylbenzene | -40 to 40 | 18 | 3.3 | 38.6 |
| butylbenzene | 5 to 40 | 9 | 1.8 | 41.5 |
| pentylbenzene | 10 to 40 | 8 | 1.8 | 41.6 |
| hexylbenzene | 10 to 40 | 8 | 4.5 | 52.1 |
| **Alkylnaphthalenes** | | | | |
| Chemical name | Temperature range (^o^C) | Number of points | SUPERTRAPP (%AAD) | Pedersen (%AAD) |
| 1-ethylnaphthalene | 0 to 40 | 10 | 5.4 | 69.2 |
| **Cycloaromatics** | | | | |
| Chemical name | Temperature range (^o^C) | Number of points | SUPERTRAPP (%AAD) | Pedersen (%AAD) |
| 1-ethyltetralin | 0 to 40 | 10 | 7.8 | 21.7 |
| **Iso-Paraffins** | | | | |
| Chemical name | Temperature range (^o^C) | Number of points | SUPERTRAPP (%AAD) | Pedersen (%AAD) |
| 2-methyloctane | 0 to 40 | 10 | 7.4 | 3.1 |
| 2-methylnonane | 0 to 40 | 10 | 32.1 | 34.5 |
| 2-methyldecane | 0 to 40 | 10 | 1.4 | 4.1 |
| 2-methylundecane | 0 to 40 | 10 | 3.0 | 6.1 |
| 2-methyldodecane | -3 to 40 | 10 | 3.4 | 10.5 |
| 2-methyltridecane | -3 to 40 | 10 | 3.1 | 16.6 |
| 2-methyltetradecane | -3 to 40 | 10 | 8.7 | 38.1 |
| 2-methylpentadecane | 0 to 40 | 10 | 18 | 58.6 |
| **N-Paraffins** | | | | |
| Chemical name | Temperature range (^o^C) | Number of points | SUPERTRAPP (%AAD) | Pedersen (%AAD) |
| nonane | -40 to 40 | 18 | 1.9 | 5.7 |
| decane | -28 to 40 | 15 | 1.6 | 6.1 |
| undecane | -8 to 40 | 11 | 1.1 | 4.2 |
| dodecane | -8 to 40 | 11 | 0.8 | 8.8 |
| tridecane | 0 to 40 | 10 | 0.3 | 11.8 |
| tetradecane | 12 to 40 | 7 | 1.1 | 18.8 |
| pentadecane | 22 to 40 | 5 | 2.3 | 25.8 |
| **Monocycloparaffins** | | | | |
| Chemical name | Temperature range (^o^C) | Number of points | SUPERTRAPP (%AAD) | Pedersen (%AAD) |
| propylcyclohexane | -40 to 40 | 18 | 7.8 | 28.7 |
| butylcyclohexane | 22 to 40 | 5 | 1.4 | 15.7 |
| pentylcyclohexane | -3 to 40 | 10 | 3.0 | 5.9 |
| hexylcyclohexane | -3 to 40 | 10 | 46.0 | 10.2 |
| heptylcyclohexane | -3 to 40 | 10 | 34.1 | 14.4 |
| octylcyclohexane | 2 to 40 | 9 | 29.6 | 25.6 |
| nonylcyclohexane | -3 to 40 | 10 | 34.5 | 29.2 |
| **Dicycloparaffins** | | | | |
| Chemical name | Temperature range (^o^C) | Number of points | SUPERTRAPP (%AAD) | Pedersen (%AAD) |
| cis-decalin | -28 to 40 | 15 | 2.2 | 28.4 |

**Table S2.** Relative reliability of the viscosity methods SUPERTRAPP and Pedersen (%AAD, Absolute Average Deviation) for molecules included in the blend model.

| **Compound** | **Concentration (g/L)** |
| --- | --- |
| Glucose | 50.09 |
| Xylose | 16.51 |
| Acetic acid | 12.58 |
| Arabinose | 0.03 |
| Lactic acid | 0.34 |
| Butyric acid | 0.00 |
| Formic acid | 0.57 |
| Furfural | 0.39 |
| Hydroxymethylfurfural | 0.00 |
| 4-hydroxybenzoic acid | 0.78 |
| Vanillic acid | 0.00 |
| Coumaric acid | 0.00 |
| Ferulic acid | 0.00 |
| Vanillin | 0.00 |
| Benzoic acid | 1.09 |

**Table S3.** Concentration of compounds tracked by HPLC in batch 2 poplar hydrolysate supplemented with 5 g/L ammonium sulfate.

**
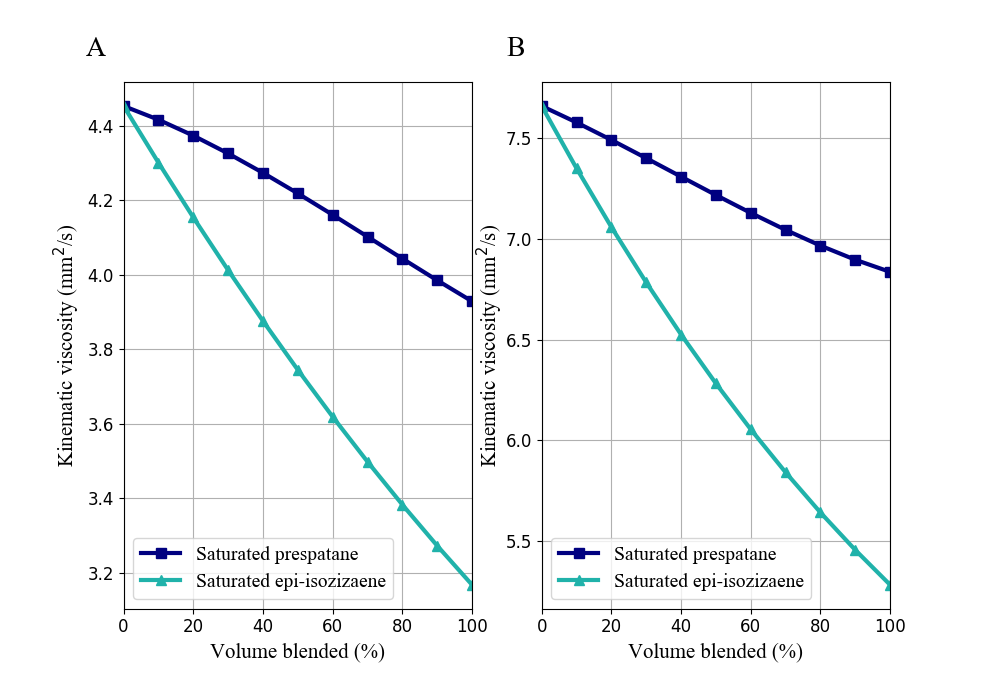
**

**Figure S1.** Viscosity blending behavior of saturated prespatane and saturated epi-isozizaene at (**A**) -20 °C and (**B**) -40 °C.

**
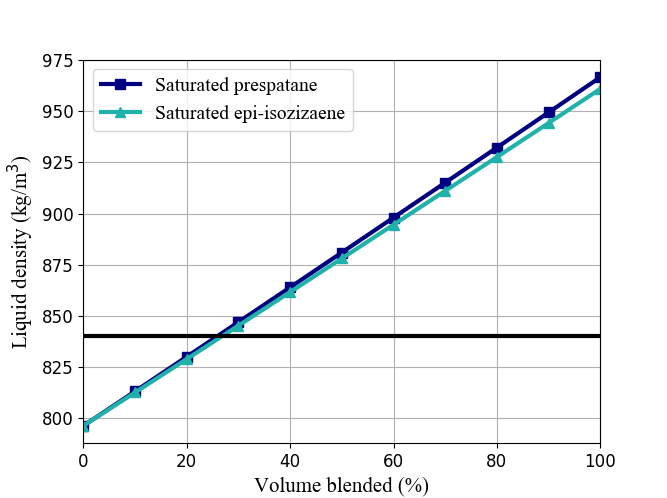
**

**Figure S2.** Liquid density blending behavior of saturated prespatane and saturated epi-isozizaene at 15 °C.

**
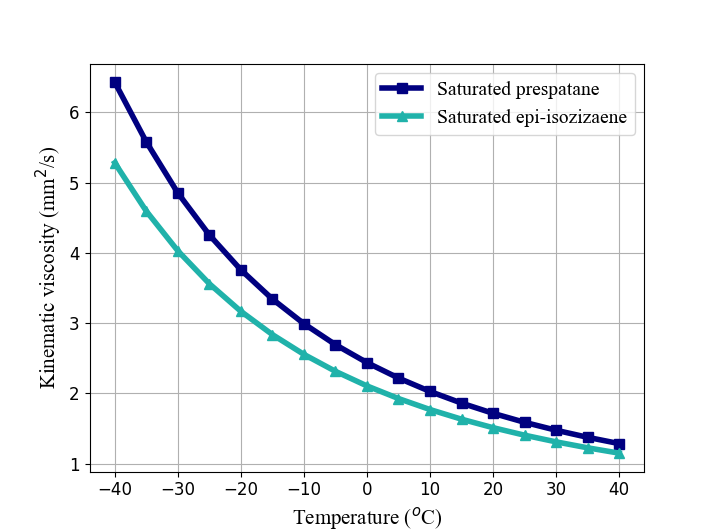
**

**Figure S3.** Viscosities of saturated prespatane and saturated epi-isozizaene, in the temperature range of -40 to 40 °C.

**Figure S4.** Sesquiterpene titers of the highest terpene producing strain for each construct shown in Figure 1, before stacking of HYG and NAT constructs. Cultures were grown in YPD_10_ with a 20% dodecane overlay. On day 7, the dodecane overlay was sampled and analyzed for epi-isozizaene and prespatane. Sample names are represented by their respective promoters: GAPDH, TEF1, and the two-promoter construct, ANT-TEF1. Native (non-codon-optimized) terpene synthases are represented by *.


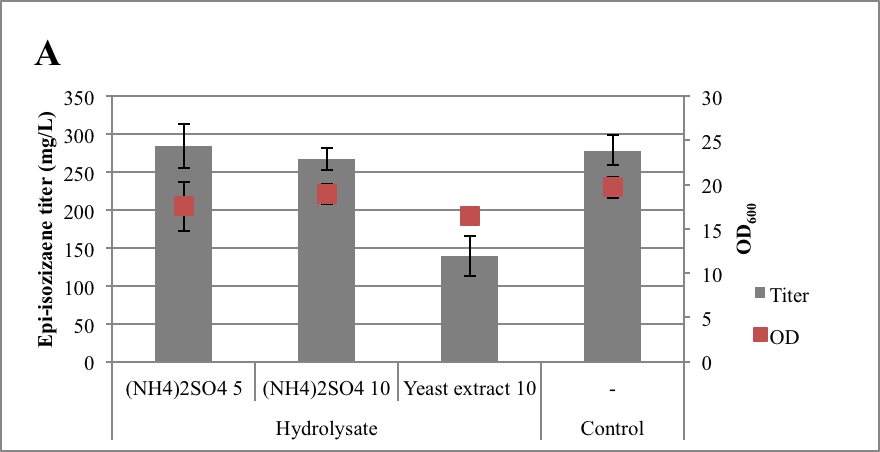

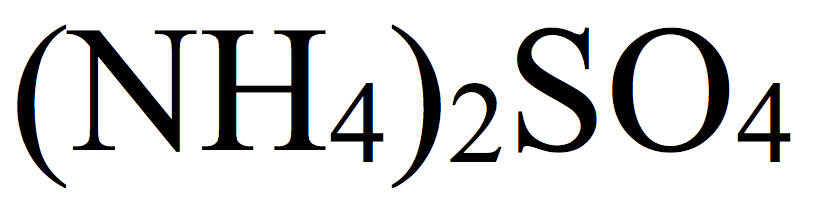

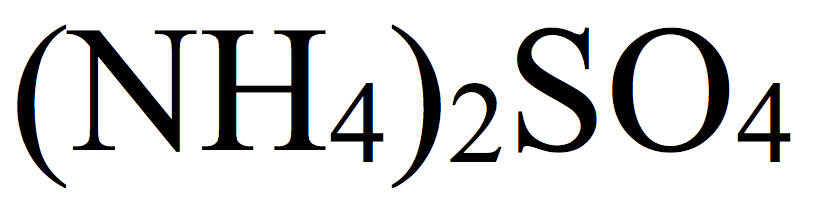


**B**

| **Medium** | **Supplement (g/L)** | **Glucose utilization (%)** | **Xylose utilization (%)** | **Epi-isozizaene titer (mg/L)** |
| --- | --- | --- | --- | --- |
| **Hydrolysate** | (NH_4_)_2_SO_4_ 5 | 99.4 ± 0.2 | 86.1 ± 0.8 | 284.0 ± 28.9 |
|  | (NH_4_)_2_SO_4_ 10 | 98.0 ± 0.9 | 88.9 ± 0.9 | 266.5 ± 14.5 |
|  | Yeast extract 10 | 98.1 ± 1.0 | 77.0 ± 0.8 | 138.9 ± 26.4 |
| **Control** | – | 100.0 ± 0.0 | – | 278.0 ± 20.0 |

**Figure S5.** Nitrogen source supplementation comparisons in poplar hydrolysate. (**A**) Epi-isozizaene titers and OD_600_ at day 7 of strain EIZS2 in filtered batch 1 poplar hydrolysate supplemented with various nitrogen sources. (**B**) Percent utilization of sugar and titer of epi-isozizaene. (n=3, data shown as average ± standard deviation, from a single experiment)

**Figure S6**. The initial 2 L fermentation run resulted in a low prespatane titer, which was attributed to a possible magnesium and phosphate deficiency. Change of titers of magnesium, phosphate, ammonium, and prespatane from strain PPS5 grown in batch 2 hydrolysate.

**Figure S7**. Organic acids from PPS5 fermentation with unfiltered hydrolysate batch 3.

**B**

| **Poplar** | **Glucose utilization (%)** | **Xylose utilization (%)** |
| --- | --- | --- |
| Filtered | 99.9 ± 0.9 | 98.9 ± 0.1 |
| Unfiltered | 36.6 ± 5.3 | 6.5 ± 1.5 |

**Figure S8.** Prespatane production by PPS5 grown in filtered and unfiltered poplar hydrolysate. (**A**) Prespatane titer and cell count for both conditions. (**B**) Utilization of glucose and xylose.


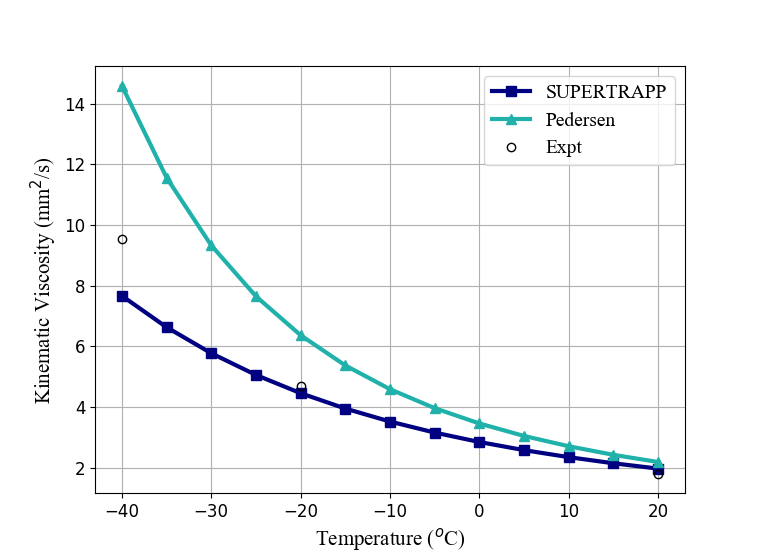


**Figure S9.** Validation of a viscosity model for Jet A. Navy (solid square) shows results from the SUPERTRAPP method, turquoise (solid triangle) shows results from the Pedersen method, and open circle indicates empirically measured viscosities.
